# Medical News and Miscellany

**Published:** 1890-11

**Authors:** 


### Medical News and Miscellany.

Read the Sanitarian's announcement. The best sanitary publication in America.

---

The Kentucky School of Medicine has over three hundred matriculants to date.

---

Attention is called to the advertisement of the Shuford Speculum. It is a "good thing."

---

Dr. W. H. Walker has removed from Shiner, in Lavacca county, to Ledbetter, Washington county.

---

Do not foget the quarterly meeting of the Austin District Medical Association in Austin on the 20th of December.

---

Proceedings of the West Texas District Medical Society and Cherokee County Medical Society received too late for this issue

**Removed.**—Dr. W. E. Fowler from Cedar Creek to Bastrop.

---

**The Journal** asks attention to Sharp & Dohme's new ad. on title page.

---

Daniel's TEXAS MEDICAL JOURNAL and the *Brooklyn Medical Journal*, both for $3.50. Address this office.

---

**Dr. C. L. Peeples**, of Navasota, and Dr. J. W. Miller, of Massey, have gone to New York to take a special course at the Polyclinic.

---

**Married**—On the 27th of May, 1890, at the residence of the bride's father, Wm. Burgess, Esq., near Pleasant Site, Ark., by Rev. B. E. Finch—Dr. Levi Mahan, of Hempstead, Texas, to Miss Lydia Burgess. The JOURNAL extends congratulations.

---

**Let Those** who contemplate joining the Texas State Medical Association at the Waco meeting in April next year not forget that it is absolutely necessary to bring their diploma. Applications for membership will not be entertained unless accompanied by a diploma.

**Removals.**—Dr. A. N. Olive has removed from Meridian to Waco. Dr. W. S. Savage has removed from Roger's Prairie to Denison. Dr. H. B. Taliaferro has removed from Bruceville to Lorena. Dr. J. M. Burleson has removed from Richland Springs to Brownwood.

---

**Death of Mrs. Dr. Skipper.**—We regret to announce the death of Mrs. Skipper, wife of Dr. R. W. Skipper, of Holley, Texas. She died in child-bed on 27th of September ult. The JOURNAL extends its sympathies to the bereaved husband. They were married November 6, 1889.

---

**Died.**—E. R. Fowler, M. D., of Bastrop, Oct. 1, 1890. Dr. Fowler was a young man, 24 years of age, and was born in Bastrop county, Texas. He graduated from the St. Louis Medical College, 1887. Dr. Fowler was an exemplary young man of great promise. The cause of death was consumption as a result of "grippe."

---

**Brutus or Cæsar!**—An esteemed correspondent calls our attention to the fact that in a recent editorial we said, "Yet Cæsar was an honorable man,"—and says we got the wrong sow by the ear; it was Brutus, of whom Mark Antony made the sarcastic remark. Thanks, Doctor, we stand corrected. We *knew* it was *one* of 'em; like the old lady and the bluing, "if it sinks it is good—or bad, one, I don't remember which."

---

**Organize.**—Gentlemen of the medical profession in Texas, where there are a sufficient number of regular physicians in any neighborhood to warrant it, you should at once organize a medical society. That is the order of the day, and the time is rapidly approaching when not to belong to the organized branch of the profession in Texas will be to be a marked man. Progress is the watch word. When organized report to the JOURNAL.

---

**Card from the Committee on Legislation.**—The gentlemen, members of the medical profession, and others who have received blanks for a petition to the Legislature, forwarded by the Committee on Legislation of the Texas State Medical Association, are urgently requested to secure as many signatures as possible, and to forward the completed lists *before the 20th of December* to Geo. Cupples, M. D., Chairman of Committee on Legislation T. S. M. A., San Antonio.

Blanks will be furnished when desired on forwarding name to address.

---

**The College of Physicians** of Philadelphia, announces that the next award of the Alvarenga prize, being the income for one year of the bequest of the Senor Alvarenga, and amounting to about one hundred and eighty dollars, will be made on July 14, 1891. Essays intended for competition may be upon any subject in medicine, and must be received by the Secretary of the College on or before May 1, 1891. CHARLES W. DULLES,

Secretary.

**Should be Encouraged.**—Dr. S. H. Stout, of Cisco, late Medical Director of Hospitals C. S. A., who some time ago called upon all surviving members of the medical corps of the Confederate States army, who served under him, to report their present whereabouts, etc., informs us that to date only three have responded. Notwithstanding this want of interest on their part he is going ahead compiling a record of their valuable services during the late war, and will publish it unaided. It is a duty he feels called on to perform even at a great sacrifice.

---

**Married.**—Thursday evening at 8 o'clock, Dr. S. L. Keown, a well known and popular physician of Comanche, led to the altar, at the residence of Major W. R. Bratton, the bride's uncle, Miss Minnie Jones, of Taylor, Texas. where the Rev. S. J. Franks, pastor of the Methodist church, in a short but impressive ceremony made them one. The Doctor and his beautiful and accomplished bride have resided long enough in Comanche to make many friends who unite in wishing them a long, useful and happy life. So mote it be.—*Comanche Ex.*

The JOURNAL extends congratulations.

---

**Remarkable Fecundity.**—J. DeLeon, M. D., Ingersoll, Texas, in the *Journal of the National Association of Railway Surgeons*, June, 1890, reports a case of quadruplets. They weighed in the aggregate nineteen and a half pounds without clothing; the first weighed six pounds; second, five pounds; third, four pounds; fourth, four pounds. The lady, Mrs. E. T. Page, is a blonde, age 36, and has given birth to fourteen children, twins three times before this. One pair by her first husband. She has been married to Mr. Page three years and has had eight children in that time. Page is an Englishman, small, dark hair, age 26, weight 115 pounds. The father in despair at the announcement of the fourth child fell across the bed and exclaimed, "Lord, God, Doctor, what shall I do?" Statistics show one quadruplets to 375,000 births. Thatthe children should all live and do well is remarkable.

The Home-Maker is the name of an excellent quarto publication issued monthly at $2 a year. It is published in New York, and is edited by Mrs. J. C. Croly, (known to all the world as "Jenny June") a lady whose experience in conducting and editing publications for the *home* probably exceeds that of any woman in the world. It has a circulation of 50,000 subscribers, and is read all over the South. It is a first-class family magazine, and well adapted to the cultivation of the mind, and morals of the young; it is beautifully illustrated.

We have made arrangements by which we can furnish the *Home-Maker* to our readers almost *free*, in connection with DANIEL'S TEXAS MEDICAL JOURNAL. Send $2.75 and we will send you this JOURNAL and the *Home-Maker* one year. An unprecedented offer. Address this office.

---

Words of Commendation.—Of the Transactions Texas State Medical Association 1890, an honored ex-President says: "The copy of the Transactions came duly to hand and well sustains the reputation of its predecessors in every respect. The Association certainly owes you a lasting debt of gratitude for your splendid work in its behalf."

A distinguished member in Falls county says: "It is issued in elegant style and I think the publishing committee deserve the highest praise."

Dr. Jno. H. Ranch, Secretary Illinois State Board of Health says: "The volume is a credit to your organization."

The Proctor of Texas University says: "As the main spring of the Association the present Secretary seems to be indispensable to its continued and orderly movement, and the Association would do well to elect him Secretary *pendente vita*."

---

Death of Dr. King.—The JOURNAL learns with much regret of the death of Dr. J. H. T. King, late of Laredo. Dr. King was the United States Consul to Mexico and was stationed at Victoria, Mexico. A telegram to Consul-General Sutton announced that Dr. King had committed suicide by poison on or about September 30. It was rumored in Laredo, says a correspondent, that he had been on a spree for a week or so prior to his death, and the sad death was perhaps the result of despondency and depression.

Dr. King was an honored member of the Texas State Medical Association, and had a fine practice, principally surgery. He leaves a wife, and a grown daughter in Baltimore. He held a diploma from the College of Physicians and Surgeons of England and stood very high in his profession.

---

**Convention of Texas World's Fair Exhibit.**—At the request of Executive Committee the President of the Texas State Medical Association has appointed the following members delegates to the convention of the Texas World's Fair Exhibit to be held in Houston December 10, 1890, (proximo).

Drs. R. M. Swearingen and F. E. Daniel, Austin; Drs. W. B. Brooks and H. K. Leake, Dallas; W. W. Reeves, Wills Point; Dr. J. F. Y. Paine, Galveston; Geo. Cupples, San Antonio; J. H. Sears, Waco; Dr. J. D. Osborn, Cleburne; Dr. E. P. Becton, Sulphur Springs; Dr. D. F. Stuart, Houston; Dr. J. B. Stinson, Sherman; Dr. C. F. Paine, Comanche; Dr. O. Fastland, Wichita Falls; Dr. B. F. Eads, Marshall, Dr. A. P. Brown, Fort Worth.

Certificates have been issued by the Secretary of the Association.

---

**Another Precinct Heard From.—Laredo Medical Society Endorses Dr. Swearingen for State Health Officer.**—Present —Drs. Arthur, McKnight, Turpin, Berg, Gorham, Wilcox and Head.

Meeting called to order by President Arthur, Dr. Turpin appointed Secretary pro tem.

The object of the meeting was stated by the President to be, to discuss the propriety of endorsing Dr. R. M. Swearingen as a candidate for State Health Officer.

Moved, seconded and carried that Dr. G. C. Head be invited to meet and act with us pending his application for membership.

WHEREAS, The time is drawing near when it will become the duty of the Governor-elect to appoint a State Health Officer, and

WHEREAS, Dr. R. M. Swearingen is of proved and tried professional ability, and a gentleman whom we all respect; therefore be it—

*Resolved*, That the Laredo District Medical Association heartily endorse Dr. R. M. Swearingen as a candidate for State Health Officer, and urge upon the Governor-elect the propriety of his appointment.

Moved and carried that a copy be sent to Governor Hogg, to DANIEL'S TEXAS MEDICAL JOURNAL and to Dr. Swearingen.

Adjourned to first Monday in December regular meeting.

J. P. ARTHUR, M. D., President.

T. J. TURPIN, M. D., Secretary.

[This was received too late to go in with similar resolutions in the department of Society Notes.—ED.]

---

**Koch's "Cure" for Consumption.**—As we write, the daily papers are printing, with every issue, some fresh news of the wonderful, and as yet secret, "cure" for consumption discovered by Dr. Robert Koch. The interest in it is natural and intense. In this country there are some two hundred thousand sufferers from this fell disease, and in Europe and other parts of the world a still greater array of victims are hopefully looking for some cure, or patiently awaiting the end.

The expectations aroused by the numerous and mysterious reports that are issued from Berlin must be very great. It is well, therefore, to consider how much can be in fact realized by the announced "cure." Of it we know at least this, that it is a germicide, and that its action depends upon its destroying the vitality or power of growth of the tubercle bacilli. If this be the case, it is a remedy that must have various limitations to its usefulness. Phthisis is, in most cases, in its inception, an infectious bacillary disease. But after lung tissue is once well invaded, and necrotic and inflammatory processes set in, the infection is a mixed one, and pyogenic organisms are largely in control. No agent which simply destroys bacilli can, therefore, seriously modify the phthisical process. Germicides cannot cure phthisis in its second and third stages.

We are satisfied that Dr. Koch will not claim this for his "cure." Already it is stated that cases of lupus, of tubercular joints, and of phthisis in the first stage have been cured. But all these forms of tuberculosis can already be relieved by surgical and hygienic measures at our disposal. It remains to be seen, therefore, whether Koch has really supplied us anything which will carry the treatment of tuberculosis any further than it already has been brought.

One thing further, perhaps, he has achieved, viz., a method of prevention for those susceptible or exposed to the disease. This may be a gain, although the prevention of phthisis in individual cases is almost always possible if proper care is taken.—*N. Y. Medical Record.*

---

**"Very Like a Whale."**—The JOURNAL has been favored with several copies of a most remarkable document, and asked to "notice" it. It is a petition to the Governor for the appointment of State Health Officer—gotten up by a Dr. J. R. Briggs, of Dallas. In the petition he says of himself that he is "a gentleman of the highest integrity and morals,"—"a physician eminent in his profession"—"a sanitarian of national reputation" and "a writer on medical and sanitary subjects who stands *pre-eminently* at the head of the medical profession in this country." (That is to say, in the United States and the Dominion of Canada, with perhaps Mexico and the West India Islands thrown in.) To this assertion some three thousand Texas physicians, we learn, have been modestly requested to give their "most cordial and unqualified endorsement."

Our correspondents have accompanied the document sent us, with remarks more forcible than complimentary to the author—and one—a gentleman resident in North Texas after asking if such methods of bringing one's self into notice which are so like those resorted to by the army of quacks which infests our State are countenanced by the State Medical Association—a body of which the petitioner is a member, proceeds to make fun of it and says: "The famous Colonel McClung, of Mississippi, when on a spree used to exclaim, 'By —! I'm a *whale!*' Little Jack Cheatham, sheriff of Boliver county, who stood four-feet-four, but brave as a lion, thought McClung's remarks on one occasion were directed to him; so, mounting a chair and gazing defiantly at the valiant and much feared Colonel, said, in his little cracked voice, 'Colonel McClung, I will have you to understand, sir, that if you *are* a whale, by George, sir, *I'm* no sardine!" '

Our correspondent suggests that Swearingen is no sardine, though he doesn't claim to be "the head of the medical profession in this country."

Another correspondent says, of the unique petition: "Old Bailey when drunk used to boast that he was a 'whale,' a 'mammoth,' a 'whole team and a spotted dog under the 'wagon;' and on one occasion he supplemented the remark with, and if there is anything on earth any bigger than *them*, I'm it!' "

If there is anything "bigger" than "head of the medical profession in this country," our correspondent suggests that the Doctor would claim to be "it" also.

